# Use a “GHOST-CAP” in acute brain injury

**DOI:** 10.1186/s13054-020-2825-7

**Published:** 2020-03-14

**Authors:** Fabio Silvio Taccone, Airton Leonardo De Oliveira Manoel, Chiara Robba, Jean-Louis Vincent

**Affiliations:** 1 Department of Intensive Care, Erasme Hospital, Université Libre de Bruxelles (ULB), Route de Lennik, 808, 1070 Brussels, Belgium; 2 Department of Critical Care, Hospital Alemão Oswaldo Cruz, São Paulo, Brazil; 3 Policlinico San Martino, IRCCS per l’Oncologia e Neuroscienze, Dipartimento di Scienze Chirurgiche e Diagnostiche Integrate, Università degli Studi di Genova, Genoa, Italy

**Keywords:** Neurointensive care, Secondary brain injury, Acronym, Management

## Background

Simple mnemonics can help prevent inappropriate or unnecessary therapeutic interventions in the ICU. Some years ago, the “FAST-HUG” acronym was developed [1], summarizing key aspects of routine ICU patient management (feeding, analgesia, sedation, thromboembolic prevention, head-of-bed elevation, ulcer prophylaxis, glucose control); this acronym is now used in many ICUs worldwide.

Management of patients with acute primary brain injury involves treatment of the primary cerebral insult (e.g., trauma, cerebral edema, tissue hypoxia, seizures) and avoidance of secondary brain injury from extra-cerebral events [2]. Hence, we propose a new acronym, “GHOST-CAP,” to help remind healthcare providers of the main factors to be considered when managing these patients.

## The GHOST-CAP components

- G: glucose is the neuron’s primary source of energy. *Hypo*glycemia (≤ 80 mg/dL) can impair brain metabolism [3] and *hyper*glycemia (≥ 180 mg/dL) has also been associated with worse outcomes [4]. In patients with acute brain injury, tight glycemic control may not significantly improve the outcomes and may increase the risk of hypoglycemia [5]. Target levels between 80 and 180 mg/dL may be reasonable (Supplemental Table 1).
- H: hemoglobin is an important determinant of oxygen delivery (DO_2_) [6]. Usually, cerebral DO_2_ is sufficient so that when cerebral blood flow (CBF) is reduced, the brain has enough physiological reserve. Although CBF can increase to preserve cerebral DO_2_, low hemoglobin levels may be associated with brain hypoxia, cell energy dysfunction, and worse outcome [7]. No well-designed randomized clinical trial (RCT) has addressed ideal transfusion thresholds in patients with acute brain injury, but a 7–9-g/dL threshold seems reasonable [6].
- O: oxygen is another important determinant of DO_2_. Hypoxemia is harmful to the injured brain, but *hyper*oxemia can be associated with excitotoxicity [8] and worse outcomes [9]. In a recent RCT, a strategy limiting oxygen exposure (i.e., target SpO_2_ 90–97%) was not associated with worse outcomes than a standard strategy in a subgroup of patients with acute brain injury [10]. Targeting a SpO_2_ between 94 and 97% seems reasonable.
- S: sodium concentration affects brain volume and is often altered in patients with acute brain injury, because of hyperosmolar fluid therapy, diabetes insipidus, inappropriate free water retention, increased natriuresis, and/or AKI. Hyper- and hyponatremia have been reported to be independently associated with worse outcomes in this patient population, and hyponatremia (sodium < 135 mEq/L) can contribute to increased brain volume and intracranial hypertension [11]. Hypernatremia may occur as a result of intracranial pressure (ICP)-directed therapies, and sodium levels up to 155 mEq/L may be tolerated in such conditions.
- T: temperature is strictly regulated to optimize cellular function. Hyperthermia is part of a systemic inflammatory reaction after acute brain injury and not usually associated with infection. Hyperthermia can be associated with increased ICP, cerebral hypoxia, metabolic distress, and worse outcomes in this setting [12]. Whether fever is a prognostic factor or a marker of severity remains unclear, but core temperatures > 38.0 °C should be avoided, particularly if associated with neurological deterioration or altered cerebral homeostasis.
- C: patient comfort, including control of pain, agitation, anxiety, and shivering, is an important goal, to avoid physical and psychological distress, excessive cerebral stimulation, increased ICP, and secondary tissue hypoxia [13]. The main aim is to keep patients calm, comfortable, and collaborative. Deep sedation may be required in some specific situations, such as elevated ICP, refractory status epilepticus, and severe shivering [13].
- A: arterial blood pressure is the main determinant of CBF. Even mild hypotension can result in brain hypoperfusion, especially in pathological conditions such as impaired cerebral autoregulation, increased ICP, cerebral edema, and/or microvascular disturbances. Achieving an “optimal” cerebral perfusion pressure (CPP) is crucial, but clinical benefits of monitoring the cerebral circulation/autoregulation need to be assessed in prospective trials. Maintaining a mean arterial pressure (MAP) ≥ 80 mmHg and a CPP ≥ 60 mmHg may be reasonable in unconscious patients; in awake patients, MAP targets can be titrated according to repeated neurological examination.
- P: acute changes in PaCO_2_ cause proportional changes in CBF (a 4% change in CBF per mmHg change in PaCO_2_). If intracranial compliance is reduced, any increase in CBF may increase cerebral blood volume, and thereby ICP. On the other hand, excessive hyperventilation can result in cerebral ischemia, and PaCO_2_ < 35 mmHg should be avoided [14].

## Individualizing the GHOST-CAP concept

The GHOST-CAP mnemonic should be used regularly, especially when changes in brain physiology occur, either spontaneously or after therapeutic interventions. Each variable should in principle be kept within “normal” ranges, but these may become inadequate in pathological conditions. For example, a “normal” MAP may be insufficient in the presence of intracranial hypertension, a “low” PaCO_2_ could be associated with cerebral ischemia particularly in the presence of brain edema, and moderate hypernatremia may be acceptable following administration of hypertonic solutions to control ICP.

**Fig. 1:**
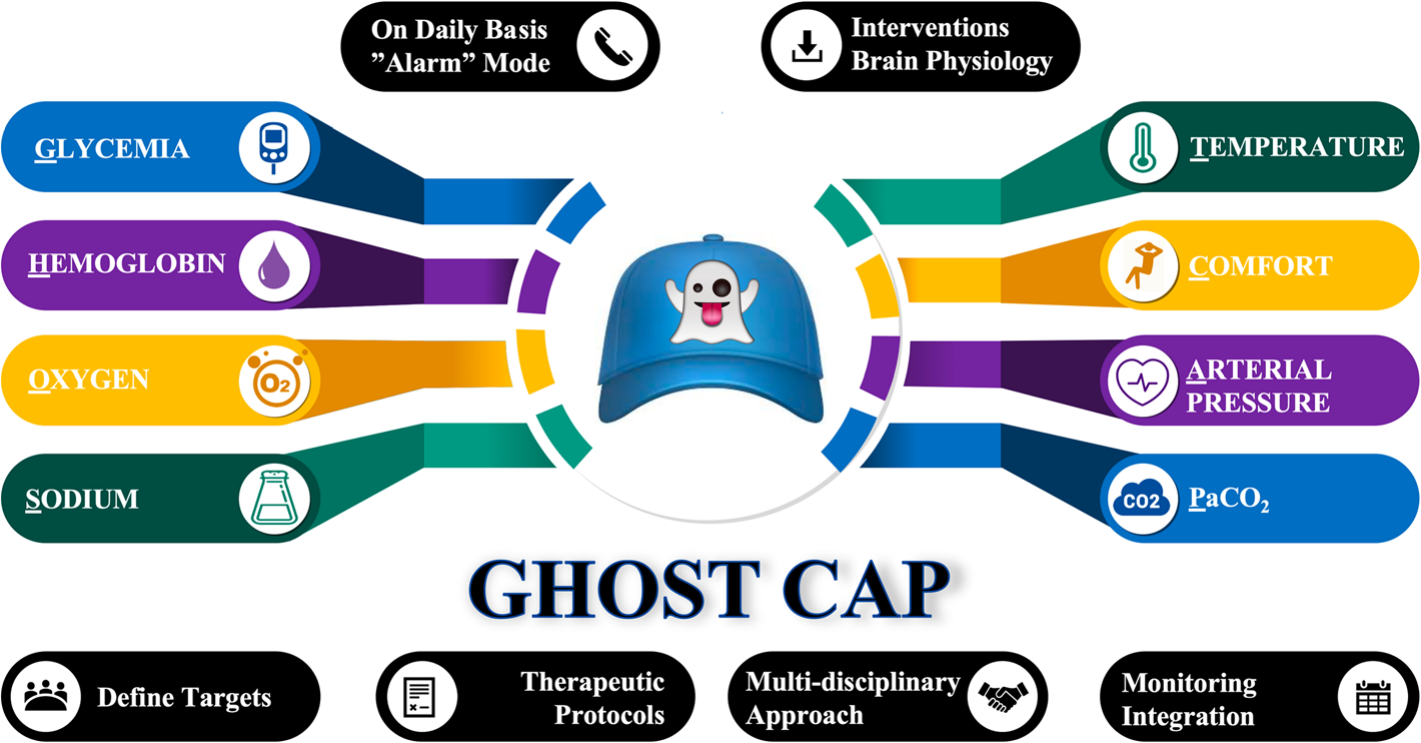
The GHOST-CAP concept

Importantly, these changes in brain physiology may not be detectable by clinical examination in unconscious patients, and optimization of each GHOST-CAP component should be guided by specific tools. The use of non-invasive monitoring (e.g., cerebral ultrasound, near-infrared spectroscopy, electroencephalography) may be challenging, and invasive tools, such as ICP monitoring, brain tissue oxygen pressure (PbtO_2_), or cerebral microdialysis (cMD), may be preferred (Fig. 1). Assessment of ICP could be useful to titrate sedation (e.g., if agitation or pain is associated with intracranial hypertension), sodium (e.g., maintain moderate hypernatremia if ICP is increased), temperature (e.g., strict normothermia if increasing body temperature increases ICP), MAP (e.g., increase in MAP resulting in an increase in ICP suggests altered autoregulation), or PaCO_2_ (i.e., to keep ICP below critical thresholds). PbtO_2_ values may be useful also to individualize PaO_2_ and hemoglobin values. Finally, cMD may be of additional interest to evaluate the metabolic changes and individualize glucose targets.

## Conclusions

The GHOST-CAP mnemonic can be easily implemented at the bedside and covers key aspects of management for patients with acute brain injury; others are included in the FAST-HUG or in specific protocols. Multimodal invasive neuromonitoring may be required to optimize target ranges and therapeutic decisions in individual patients. We believe this concept could encourage teamwork and improve quality-of-care.

## Supplementary information

**Additional file 1: Supplemental Table 1.** The GHOST-CAP mnemonic with reasonable target values for each component and when specific invasive monitoring systems could help individualize the targets.

## Data Availability

Not applicable

## Abbreviations

AKI: Acute kidney injury
PbtO_2_: Brain oxygen pressure
CBF: Cerebral blood flow
cMD: Cerebral microdialysis
CPP: Cerebral perfusion pressure
ICP: Intracranial pressure
ICU: Intensive care unit
MAP: Mean arterial pressure
DO_2_: Oxygen delivery
SpO_2_: Oxygen saturation
RCT: Randomized clinical trial
