# Supplementary material for: Use a “GHOST-CAP” in acute brain injury
Source: Crit Care. 2020 Mar 14;24:89. doi: 10.1186/s13054-020-2825-7 (PMC7071769; doi:10.1186/s13054-020-2825-7)
Supplement: Supplementary file 1 — Additional file 1: Supplemental Table 1. The GHOST-CAP mnemonic with reasonable target values for each component and when specific invasive monitoring systems could help individualize the targets. [file 13054_2020_2825_MOESM1_ESM.docx]

**Supplemental Table 1.** The GHOST-CAP mnemonic with reasonable target values for each component and when specific invasive monitoring systems could help individualize the targets.

|  |  | **Targets** | **ICP** | **PbtO_2_** | **cMD** |
| --- | --- | --- | --- | --- | --- |
| **G** | **Glucose** | 80 - 180 mg/dL |  |  | x |
| **H** | **Hemoglobin** | > 7 g/dL |  | x |  |
| **O** | **Oxygen** | SaO_2_ = 94-96%  PaO_2_ = 80-120 mmHg |  | x | x |
| **S** | **Sodium** | 135 – 145 mEq/L | x |  |  |
| **T** | **Temperature (core)** | < 38.0°C | x | x | x |
| **C** | **Comfort** | No pain and agitation | x | x |  |
| **A** | **Arterial pressure** | MAP > 80 mmHg  CPP > 60 mmHg | x | x | x |
| **P** | **PaCO_2_** | 35 - 40 mmHg | x | x | x |

MAP = mean arterial pressure; CPP = cerebral perfusion pressure; ICP = intracranial pressure; PbtO_2_ = brain oxygen pressure; cMD = cerebral microdialysis
